# Single group study to evaluate the feasibility and complications of radiofrequency ablation and usefulness of post treatment position emission tomography in lung tumours

**DOI:** 10.1186/1477-7819-2-30

**Published:** 2004-09-06

**Authors:** Shijun Kang, Rongcheng Luo, Wangjun Liao, Hubing Wu, Xuelin Zhang, Yuru Meng

**Affiliations:** 1 Department of Oncology, Nanfang Hospital, Guangzhou, P.R.China, 510515; 2 PET Center, Nanfang Hospital, Guangzhou, P.R.China, 510515; 3 Medical Image Center, Nanfang Hospital, Guangzhou, P.R.China, 510515

## Abstract

**Background:**

There is genuine need to develop interventional treatment options for management of lung tumors. Radiofrequency ablation (RFA) is one such alternative being promoted to treat lung tumors recently. Larger studies should help define RFA's further development. Furthermore fluorodeoxyglucose positron emission tomography (PET) has been reported to be an accurate indicator of treatment response in variety of tumors. This study focuses on the evaluating the feasibility of RFA and usefulness of PET scan in lung tumors after RFA procedure.

**Patients and methods:**

Between November 1999 and May 2002, 50 patients with primary or metastasis pulmonary tumors underwent RFA procedure. The electrode was guided to the target areas using computerized tomography (CT). Tumors smaller than 3.5 cm were given single RFA, while tumors larger than 3.5 cm received RFA to multiple sites. Maximum 4 lesions or 6 target areas were treated during one operating procedure. Whole body and/or lung PET images were acquired; identical site CT images and chest X-ray were taken 1 week before and after RFA.

**Results:**

Of the 50 patients, 17 had single lesions while rest had multiple lesions. Tumors smaller than 3.5 cm were completely dissipated after RFA. In tumors larger than 3.5 cm, the part within 3.5 cm diameter dissipated. While CT showed that tumor image became larger 1 to 2 weeks after RFA procedure. PET demonstrated tumor destruction in 70% cases, compared to 38% in CT.

**Conclusion:**

The present study shows RFA to be safe and effective treatment option for lung tumors. PET is superior to CT in evaluation the effectiveness of RFA treatment shortly after the procedure.

## Background

Lung cancer continues to be the leading cause of cancer deaths in United States [1]. The overall prognosis of lung cancer is still dismal despite all current early detection and treatment efforts. Only about 20–25% of lung cancers can potentially be cured by surgery. The majority of patients presents with locally advanced or metastatic disease, and treatments essentially rely on external beam irradiation, chemotherapy or a combination of both [2]. Thus other interventional palliative treatment options have been developed for these lesions.

Radiofrequency ablation (RFA), is an imaging-guided percutaneous ablative procedure, that has been suggested to be an effective treatment option for patients with non-small cell lung cancer (NSCLC) and metastatic disease who are not suitable candidates for surgery [3,4]. Guided by computed tomography (CT), physicians are able to localize the tumor and determine the optimal approach. During RFA, current passing through tissue from the active electrode leads to ion agitation and frictional heat generation. This leads to irreparable cellular damage and coagulation necrosis [5]. Recently a number of studies reported its application in malignant lung tumours. Accurate assessment of treatment response remains one of the major problems.

PET has been reported to be an accurate indicator of treatment response in variety of tumors [13-17]. However, its use has been limited to evaluating disease stage in lung tumors [18-24]. PET imaging, provides proliferation and metabolism information, is sensitive and specific to diagnose malignant lesions from benign. Coleman and colleagues has provided substantial information in evaluating the role of PET in management of lung cancers [21-23]. In this report we focus on evaluating the feasibility of RFA, its complication and on evaluating the role of PET on RFA response in lung tumors.

## Patients and methods

Between November 1999 and May 2002, 50 patients with either primary or metastatic lung tumors were enrolled in to a prospective single group trial. Patient characteristics are detailed in Table 1. Patients with bleeding potentials or serious heart, liver and renal failures were excluded. Antibiotics and medicines for prevention of bleeding were given regularly. Every patient underwent a chest Flurodeoxyhlucose postron emission (PET) and CT scan before procedure.

**Table 1:** Patient characteristics

	Patients (n = 50)		
Patient characteristic	No		%
Age, years			
Median		51	
Range		35–74	
Sex			
Male	32		64
Female	18		36
Origins			
Primary lung tumors	23		46
Metastases from breast	13		26
Metastases from colon	9		18
Metastases from other places	5		10
No. of patient with lesions			
Single lesion	17		34
Multiple lesions	33		66
Total lesions received RFA		120	

Patients received a chest X-ray and CT for preoperative evaluation and a repeat scan after RFA procedure. A PET scan was performed one week after the treatment.

The Radiofrequency ablation was carried out using RF-2000 generator and related software purchased from Radio Therapeutics Corporation, USA; PET imaging was done using an Advance 2 Scanner (General Electric Medical Systems, WI, USA).

Patients received general anesthesia along with local infiltration of Lidocaine. The electrodes were directed to target areas during RFA procedure using CT scan. The initial power applied was 50 W, which was subsequently increased to a maximum 90 W over several minutes. RFA continued for 5 to 15 min until roll off was achieved, which continued for 2 min to stop. Tumors smaller than 3.5 cm were given full heating energy only once, while tumors larger than 3.5 cm received multiple RFA to different areas. Maximum 4 lesions or 6 target areas were treated during one procedure.

One to two weeks after the procedure and a 4 hour fast, patients were taken for PET scan. They were made to rest for 15 min, and then received ^18^F-FDG 296 MBq – 440 MBq (8 mCi -12 mCi) intravenously. After another period of rest lasting for 45–60 min, the whole body and/or lung images was acquired by PET scanner. PET was also acquired at 5–8 bed positions, typically from the base of skull to the mid thigh, which was identical to the CT protocol used in the present study. The complications of the treatment are detailed in table 2 and results are summarized in table 3.

**Table 2:** Complications of Radiofrequency ablation

	Patients (n = 50)	
Complication	No	%
Fever	10	20
Congested pneumonia	6	12
Pneumothorax	9	18
Hemothorax	1	2

**Table 3:** Early effectiveness of RFA by various techniques

	Tumor destruction demonstrated	
Technique	No	%
All 50 patients received		
PET	35	70
CT	19	38
X-ray	13	26

## Results

After RFA procedure a number of complications were seen. Fever and/or congested pneumonia were commonest complications seen in 32% of patients; however, they were cured in a week with antibiotics treatment. Pneumothorax occurred during procedures in 18% and the patients were treated with aspiration. Five of these had small pneumothorax that did not require and treatment. One patient had hemothorax which required intercostals drainage (ICD) which was removed 2 days later. These were no life threatening events or deaths.

**Figure 1:**
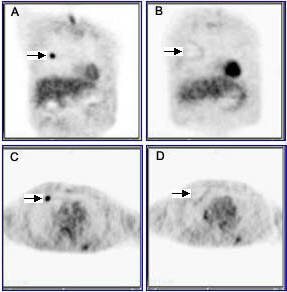
PET images taken before and after RFA treatments: coronal (A, B), and Sagittal (C, D) views of PET scans of lung cancer. (A, C) were taken before RFA treatment; (B, D) were taken two weeks after RFA treatment.

**Figure 2:**
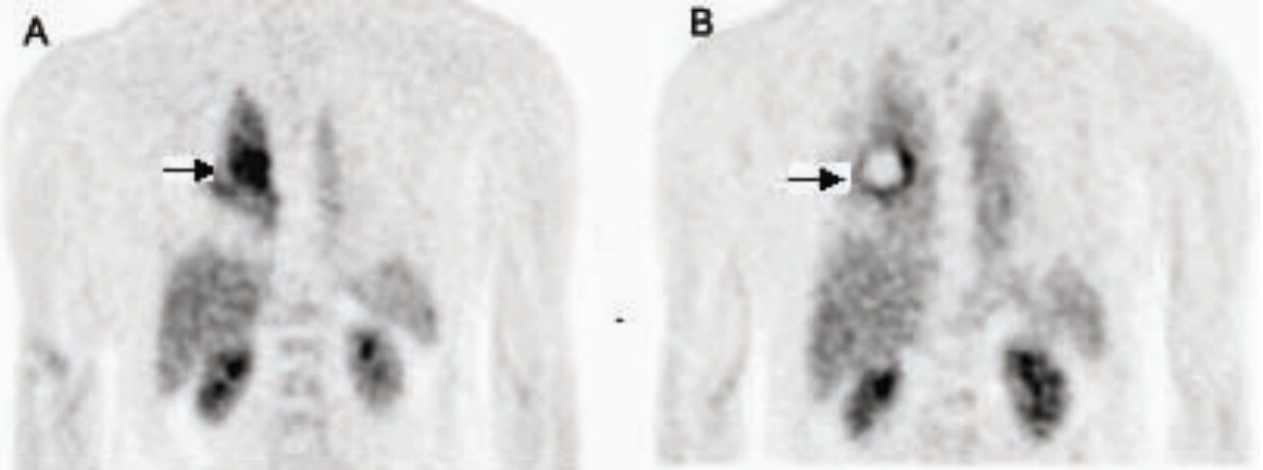
PET images taken before and after RFA treatments. This patient had a tumor size larger than 3.5 cm. PET scans were taken 1 week before (A) and 2 weeks after (B) RFA treatment.

Post procedural PET demonstrated the effectiveness of RFA on lung tumors. Tumors smaller than 3.5 cm showed complete response after RFA (Figure 1). In tumors larger than 3.5 cm, the part within 3.5 cm diameter dissipated, while the part outside this 3.5 cm area remained (Figure 2). Damage to the normal tissue outside the tumor was not extensive in any cause.

**Figure 3:**
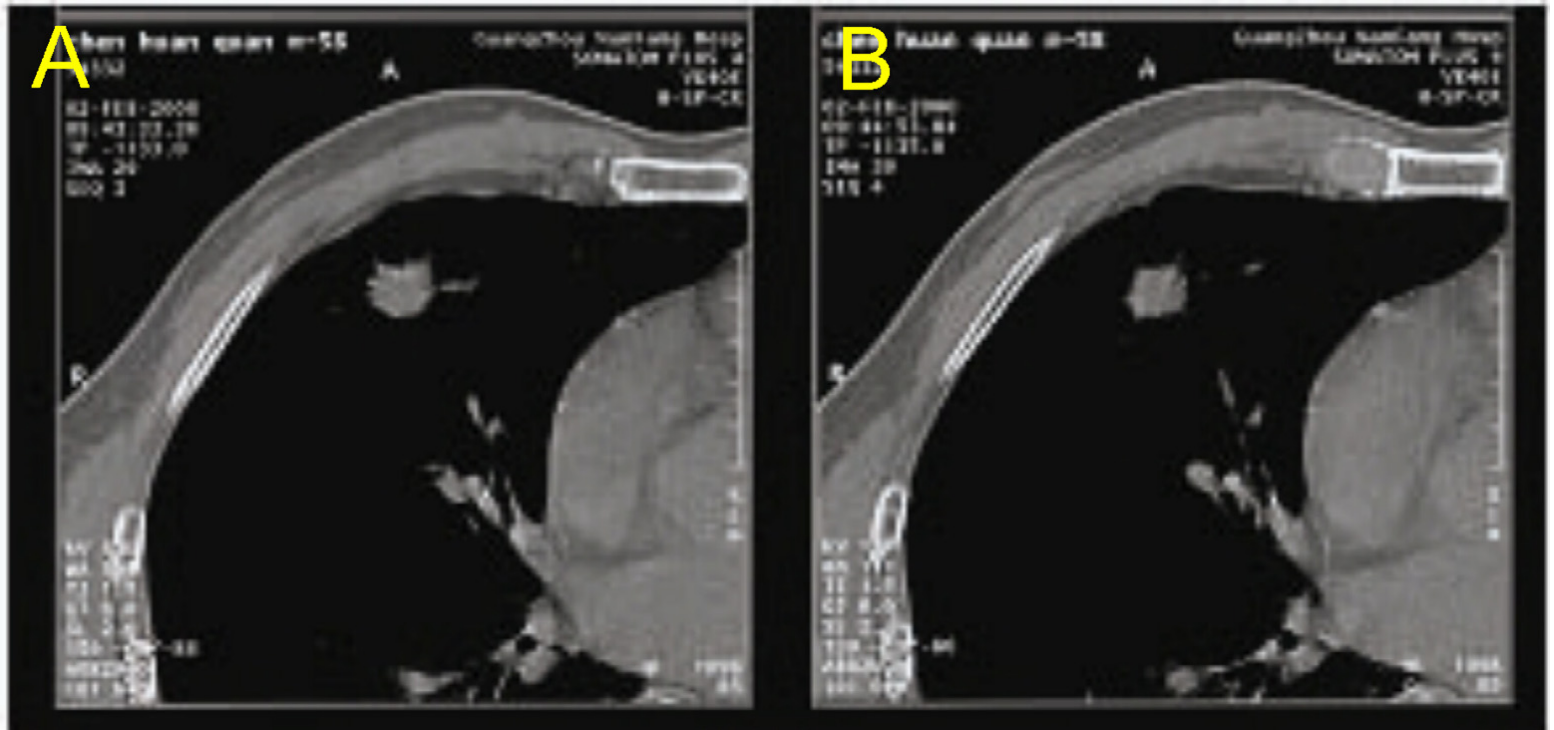
CT images taken before and after RFA treatments. The same patient PET images were shown as Figure 1. (A) was taken before RFA treatment, (B) was taken 2 week after RFA treatment.

The Chest X-ray and CT showed that tumor image became larger 1 to 2 weeks after RFA procedure (Figure 3). These may result from partial tissue damages, bleeding, acute inflammation or pneumonia, and support the routing use of antibiotics and haemostatic drugs after RFA. The tumor destruction was picked up by PET much effectively when compared to CT scan or chest X-ray.

## Discussion

Since RFA ablates lung tumors directly and locally, marginal tissues surrounding the tumor are frequently partially damaged leading to occasional pneumonia. It is difficult for regular CT and/or chest X-rays to discriminate pathological-physiological tissue damage and fibrillation from the treatment effect of RFA. PET on the other hand provides information on functional and metabolic activity anatomically, and is the only available technique which can specifically diagnose tumors or necrosis after surgery and radiotherapy effectively [28]. Our experience too proves that PET is particularly superior to CT in its ability to evaluate the effectiveness of RFA treatment early after therapy.

RFA is a relatively noninvasive, well-tolerated approach. It could destruct tumor completely within the effective diameter while avoiding the surgery, side effects of radiotherapy and toxicity of high dose chemotherapy. Our observations suggest that RFA can kill lung tumors smaller than 3.5 cm after a single RFA procedure. The effect of RFA appears to be limited within 3.5 cm diameter area with the current instruments. However, this also suggests that RFA may not damage the normal tissues surrounding the small tumors. The malignant lesions dissipated in 1 to 2 weeks, while the surrounding tissue stayed intact. While at this period regular chest CT and chest X-ray may show enlarged lesion images. This is in agreement with other reports. With improvements in technology, RFA in combination with other options may further reduce the morbidity and mortality of cancer deaths [11]. Though complications do occur, they are usually curable. RFA results in a higher rate of complete necrosis and requires fewer treatment cycles compared to traditional chemotherapy or radiotherapy. Besides CT guidance help to localize the tumor and determine the optimal approach further optimizes specific of targeting the tumor. For patients with non-small cell lung malignancy who are not candidates for surgery owing to poor cardio respiratory reserve, RFA alone or followed by conventional radiation therapy or chemotherapy may prove to be a treatment option [11]. For patients with metastatic disease, RFA may be suitable for treatment of a small tumor or reduce symptoms caused by large tumor burden. This technique can be used as a primary technique or in conjunction with other interventional procedures [11]. Further randomized controlled trials comparing RFA with conventional palliative treatment are needed before RFA can be accepted as a routine treatment modality. Survival of patient and quality of life issues too need be addressed.

## Conclusions

Despite inherent deficiency of trial design our single group study clearly demonstrates that RFA can be an effective treatment option for lung tumors. Unlike other interventional techniques, RFA provide controlled regions of coagulation necrosis with a single application to an area with 3.5 cm diameter. RFA may cure small lung tumor, reduce tumor burden in larger lesions and may be combined with external beam radiation and/or systemic chemotherapy for further improvements. PET provides functional and metabolic activity anatomically and is particularly superior to CT in evaluation the effectiveness shortly after RFA procedure. Absence of follow-up information and randomization in the current study are two major fallacies which need to be addressed in subsequent studies.

## Authors' contributions

SJK is the leading physician and drafted the manuscript.

RL, WL, HW, XZ, YM all participated in the study, patient management, literature search and preparation of manuscript.

All authors have read and approved the final version of the manuscript.

## Competing interests

None declared.
